# A Novel Nomogram Combined the Aggregate Index of Systemic Inflammation and PIRADS Score to Predict the Risk of Clinically Significant Prostate Cancer

**DOI:** 10.1155/2023/9936087

**Published:** 2023-01-12

**Authors:** Wenliang Xie, Zifan Xu, Yifan Qiu, Wei Ye, Zhiyu Zhang, Chao Wang, Jin Zang

**Affiliations:** ^1^Department of Urology, The First Affiliated Hospital of Soochow University, Jiangsu, Suzhou 215006, China; ^2^Key Laboratory of Tumor Immunology and Microenvironmental Regulation of Guangxi, Guilin Medical University, Guilin, 541004 Guangxi, China

## Abstract

**Background:**

This study is aimed at constructing a nomogram to predict the risk of clinically significant prostate cancer (csPCa) based on the aggregate index of systemic inflammation (AISI) and prostate imaging-reporting and data system version (PIRADS) score.

**Methods:**

Clinical data on patients who had undergone initial prostate biopsy from January 2019 to December 2021 were collected. Patients were randomized in a 7 : 3 ratio to the training cohort and the validation cohort. Potential risk factors for csPCa were identified by univariable and multivariate logistic regression. Nomogram was conducted with these independent risk factors, and calibration curves, the receiver operating characteristic (ROC), and decision curve analysis (DCA) were employed to assess the nomogram's ability for prediction.

**Results:**

A total of 1219 patients were enrolled in this study. Multivariate logistic regression identified that age, AISI, total prostatic specific-antigen (tPSA), free to total PSA (f/tPSA), prostate volume (PV), and PIRADS score were potential risk predictors of csPCa, and the nomogram was developed based on these factors. The area under the curve (AUC) of the training cohort and validation cohort was 0.884 (95% CI: 0.862-0.906) and 0.899 (95% CI: 0.867-0.931). The calibration curves showed that the apparent curves were closer to the ideal curves. The DCA results revealed that the nomogram model seemed to have clinical application value per DCA.

**Conclusion:**

The nomogram model can efficiently predict the risk of csPCa and may assist clinicians in determining if a prostate biopsy is necessary.

## 1. Introduction

Prostate cancer (PCa) is the second most frequently diagnosed cancer and the fifth most prevalent cause of cancer-related mortality in men worldwide, seriously threatening men's life [1]. PCa progresses very slowly and usually has no apparent symptoms in the early stage [2]. PSA is an essential clinical indicator for early detection of prostate cancer, and a prostate biopsy is recommended when abnormal PSA levels or rectal exam are detected [3]. However, diseases such as urinary tract infection, acute prostatitis, and benign prostatic hyperplasia may cause an elevation of PSA levels, which may easily lead to an excessive prostate biopsy [4]. Meanwhile, prostate biopsy as an invasive operation will inevitably bring some complications, including bleeding, infection, and pain [5]. Therefore, it is essential to explore susceptible and specific indicators for the early diagnosis of PCa.

It is believed that the development of tumors is influenced by the interaction between systemic inflammation and the local immune response [6]. Indeed, inflammatory cells and proinflammatory mediators consistently increase in peripheral blood before cancer diagnosis and may promote cancer development [7]. Cancer-associated inflammation includes cytokines, immune cells, and inflammatory protein mediators. Immune cells are mainly neutrophils, monocytes, lymphocytes, and platelets. Recently, a combination of these systemic inflammatory parameters including neutrophil-lymphocyte ratio (NLR), platelet-lymphocyte ratio (PLR), systemic immune-inflammatory index (SII), and AISI have been reported to be significant predictors of certain malignant solid tumors [8–11]. AISI is a composite index based on lymphocyte, neutrophil, monocyte, and platelet counts. However, the diagnostic value of AISI for csPCa has not been reported so far.

Compared to other routine imaging examination, multiparametric magnetic resonance imaging (mpMRI) shows the more advanced diagnostic efficacy in the diagnosis of prostate cancer [12], and the prostate imaging-reporting and data system (PIRADS) based on 3.0T mpMRI was proven to be more accurate in determining the localization, diagnosis, and risk grouping of prostate cancer, even more specific and reliable than systematic biopsy in the diagnosis of csPCa [13, 14].

As a commonly used tool in the field of oncology research [15–17], nomograms are perfectly capable of converting complex regression equations into visual graphs, providing more readability quality in terms of the clinical outcome of predictive models and exerting the tremendous facilitation for the doctors to evaluate [18] and the intuitive and identifiability for which nomograms have gained the increasing popularity and application in medical research and clinical practices. This study is aimed at investigating the diagnostic value of inflammatory indicators and PIRADS score in patients with csPCa and to construct a risk nomogram model.

## 2. Materials and Methods

### 2.1. Patient Characteristics

**Figure 1:**
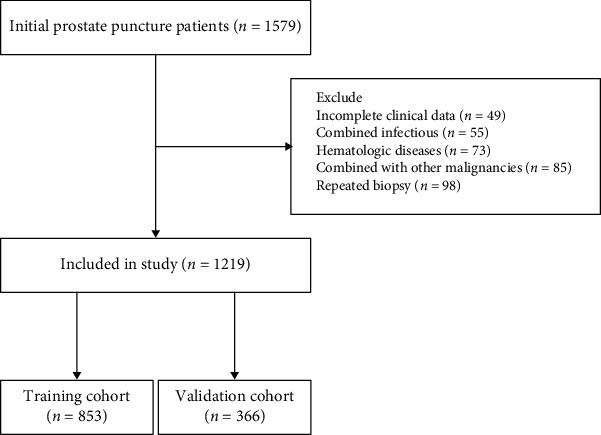
Flow chart of patient selection.

Retrospective data were collected on 1579 patients with initial prostate biopsies performed at the First Affiliated Hospital of Soochow University between January 2019 and December 2021. The exclusion criteria were patients with (a) combined infectious or hematologic diseases, (b) coagulation dysfunction, (c) combined with other malignancies, (d) repeated biopsy, and (e) incomplete clinical data. Patients were randomized in a 7 : 3 ratio to the training cohort and the validation cohort (Figure 1). The project was approved by the Ethics Committee of the First Affiliated Hospital of Soochow University.

### 2.2. Hematology Analysis

Collection of fasting venous blood was performed in the early morning within one week before prostate biopsy, including tPSA, free PSA (fPSA), platelet counts, neutrophil counts, lymphocyte counts, and monocyte counts. F/tPSA = fPSA/tPSA. Inflammatory composite index was calculated from NLR = neutrophil counts/lymphocyte counts, PLR = platelet counts/lymphocyte counts, SII = neutrophil counts × platelet counts/lymphocyte counts, and AISI = neutrophil count × monocyte counts × platelet counts/lymphocyte counts.

### 2.3. Statistical Analysis

The continuous variables following normal distribution were presented as means ± standard deviation, and the *t*-test was used to assess differences between groups. The continuous variables deviating from normal distribution were expressed as medians and ranges, and the Mann–Whitney *U* test was used to compare the differences between groups. Categorical variables were defined as the number of cases and composition ratio, and the Chi-square test was used to compare different groups. Multivariate logistic regression was used to determine independent risk factors, which were further entered into the nomogram construction. The nomogram was drawn by R software. Internal validation of the model was tested using 1,000 bootstrap resamples, and the calibration plot was showed graphically. Clinical utility was assessed via decision curve analysis (DCA). Receiver operator characteristic (ROC) curves were performed, and the area under the curves (AUC) was compared to evaluate the utility of the risk model.

## 3. Results

### 3.1. Clinical Characteristics

A total of 1219 patients were finally included in this project based on the exclusion criteria, including 853 patients in the training cohort and 366 patients in the validation cohort. Except for hypertension, there was no significant difference between the training and validation cohorts regarding baseline characteristics (Table 1). Of the 853 patients in the training cohort, 455 patients were diagnosed with PCa (53.3%), while 406 patients were confirmed as csPCa (47.6%). Compare to those without PCa, older age, higher AISI, SII, NLR, tPSA, and fPSA, less f/tPSA, smaller PV, and more PIRADS score were shown in patients with PCa (Table 2). These differences were also present in the no csPCa and csPCa groups (Table 3).

### 3.2. Establishment and Verification of the Nomogram Model

**Figure 2:**
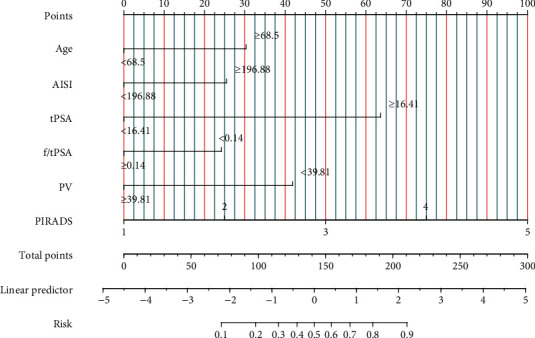
Nomogram for predicting the risk of csPCa. Instructions: the risk factors for each patient were shown on a variable axis in this nomogram, and the number of points each risk factor received was calculated by drawing a vertical line upward.

**Figure 3:**
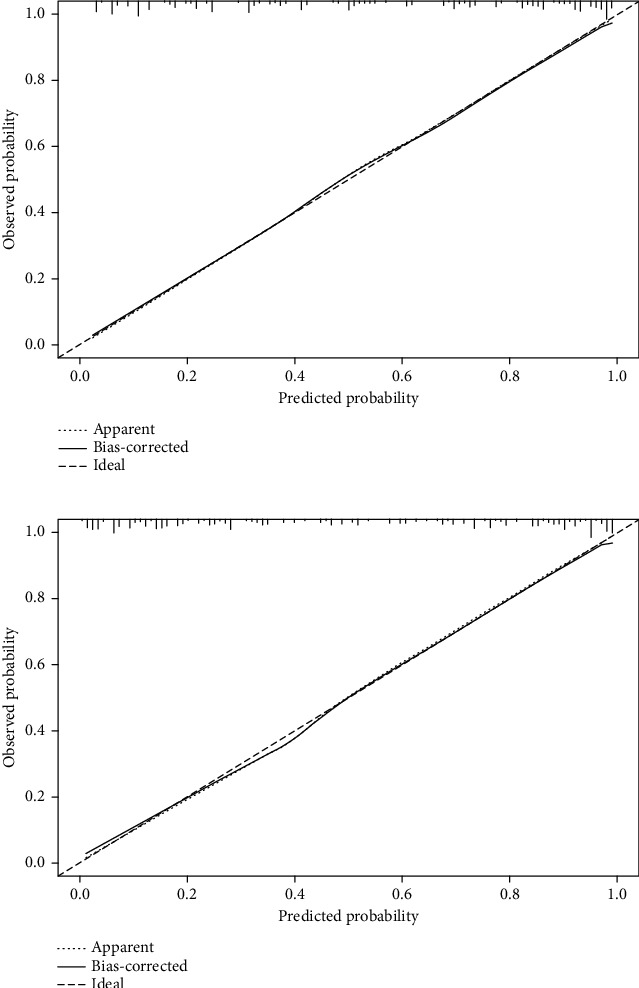
Calibration curves in the training and validation cohorts (a, b). The *x*-axis shows the model's predicted probability, and the *y*-axis shows the actual probability.

**Figure 4:**
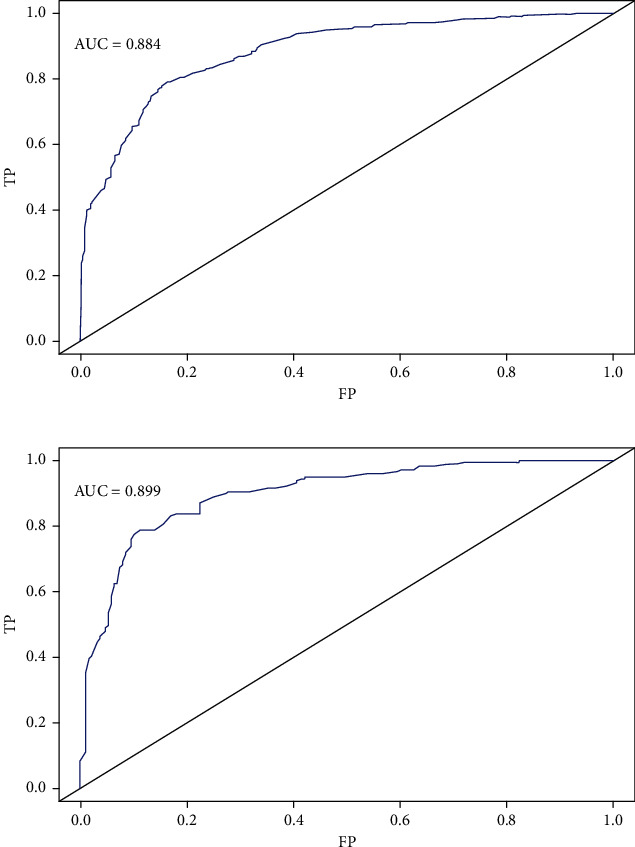
Comparison of the diagnosis value of the nomogram by the ROC curves in the training and validation cohorts (a, b). Abbreviations: FP: false positive; TP: true positive.

**Figure 5:**
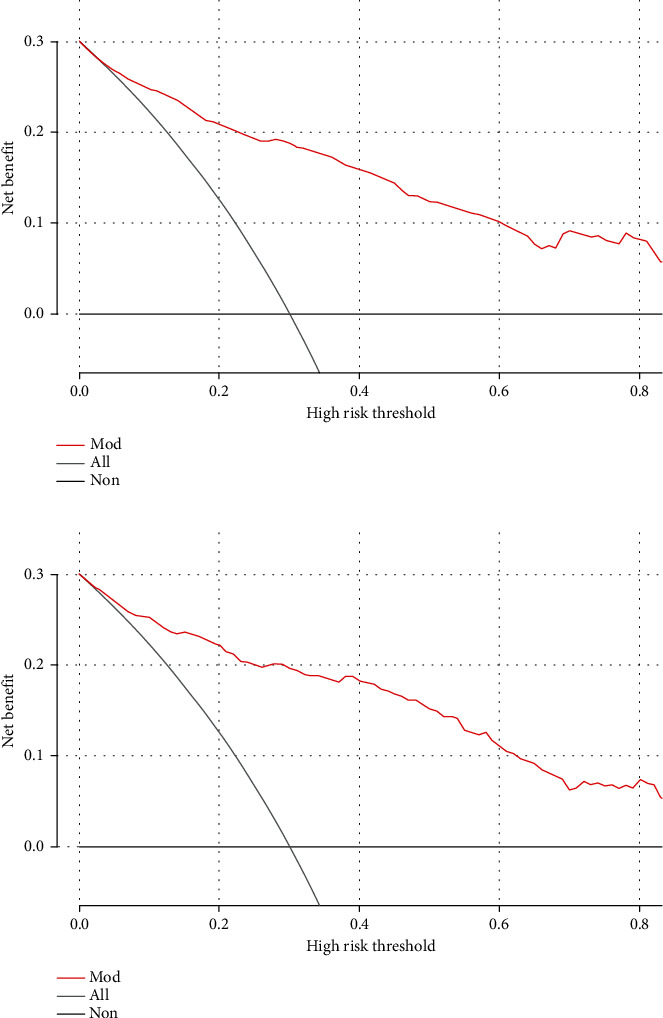
Decision curve for prediction of the risk of csPCa. Gray line: assume all patients will have csPCa; Black line: assume no patient will have csPCa; Red line: binary rule based on nomogram model.

The results of univariable and multifactorial logistic regression analysis showed that age, AISI, tPSA, f/tPSA, PV, and PIRADS score were independent risk factors for csPCa (Table 4). The data of ROC curve analysis illustrated that the addition of the AISI and PIRADS score increased the AUC of the model based on baseline variables (age, tPSA, f/Tpsa, and PV) from 0.861 (95% CI 0.836−0.886, *P* < 0.001) to 0.901 (95% CI 0.881−0.921, *P* < 0.001) in predicting csPCa (Table 5). The above independent predictors were integrated to develop a nomogram predicting csPCa (Figure 2). The calibration curves demonstrated good consistency between the predicted and observed probabilities in both the training and validation cohorts (Figure 3). In addition, the ROC analysis revealed that the AUC was 0.884 (95% CI: 0.862-0.906) for the training cohort and 0.899 (95% CI: 0.867-0.931) for the validation cohort (Figure 4). As shown in Figure 5, the DCA is based on continuous potential risk thresholds, showing the model's therapeutic usefulness by presenting the net benefit of risk-stratifying patients. The DCA curves of both the training and validation cohorts demonstrated that increase in clinical benefits during almost all threshold probabilities.

## 4. Discussion

In this study, we confirmed the correlation between AISI and the occurrence of csPCa. Based on AISI and 5 other clinical parameters, we constructed a nomogram to predict the probability of csPCa occurrence. The validation of the nomogram showed its good discriminatory and calibration ability.

Tumor formation, metastasis, and host antitumor immunity are all significantly influenced by inflammation [19]. The combination of neutrophils, monocytes, platelets, and lymphocytes comprehensively reveals the relationship between cancer cells and systemic immune inflammation [20]. Neutrophils were believed to increase DNA instability by producing and releasing toxic DNA substances [21]. Neutrophils release nitric oxide synthase during chronic inflammation, damaging DNA and leading to cellular mutations in vitro [22]. In addition, MMP-9 released from neutrophils promotes the release of vascular endothelial growth factors and angiogenesis [23]. Malignant cells form only a tiny part of the tumor ecosystem. The vascular system, lymphatic vessels, stromal compartments, and extracellular matrix are critical noncellular components of the tumor microenvironment. Monocytes and monocyte-derived cells can shape many microenvironmental features to promote tumor growth [24]. Platelets store prominent growth factors in their alpha granules [25]. Platelet-derived growth factor, transforming growth factor, and vascular endothelial growth factor are all secreted by activated platelets [26]. These growth factors stimulate angiogenesis and tumor neovascularization and induce tumor growth [27]. Lymphocytes can inhibit tumorigenesis by causing cytotoxic cell death and cytokine secretion and inhibiting tumor cell migration and proliferation [28]. Furthermore, low lymphocyte counts are linked to poor prognosis for tumor patients, probably because the host's immunity against cancer is weakened as lymphocyte levels decrease [29].

Blood cell counts are widely utilized and available in clinical practice as the most common tool to reflect systemic inflammatory response. Systemic inflammatory markers such as NLR, PLR, SII, and AISI generated from the inflammatory cell counts are independent predictors of various cancers. In our study, these four blood-related composite markers were selected and further screened to construct a nomogram by AISI. AISI was computed based on the number of platelets, neutrophils, lymphocytes, and monocytes, which can represent most blood cell types. We found that AISI may be a potential marker to predict the risk of csPCa development. Furthermore, we hope that AISI can be used in combination with other biomarkers as a valuable indicator to assess the risk of prostate cancer and provide some help to improve the accuracy of biopsy in patients.

Apart from the acknowledged systemic inflammation, one of the most significant pathological changes is the localized inflammation, which also plays an important part in the development of prostate cancer. Bacterial prostatitis, as a common urinary system disease, accounts for approximately 5-10% of the overall prostatitis cases [30]. In addition to affecting the patient's quality of life, bacterial prostatitis will also inevitably cause fluctuations in PSA levels and blood cells, decreasing the accuracy of making the precise diagnosis of prostate cancer [31], while the failure to include acute or chronic prostatitis in the study of this paper may somewhat affect the objectivity and referential of this predictive model.

Age and prostate volume are fundamental clinical parameters in most prostate cancer studies. Cormio et al. [32] developed a prediction model based on age, prostate volume, etc. The AUC of this model was 0.800 which was lower than us (AUC 0.884 [95% CI 0.862-0.906, *P* < 0.001]). Moreover, the prostate cancer specific biomarkers are widely used in clinical studies [32–34]. Ploussard et al. [35] conducted a prospective study based on 667 individuals, and the efficacy of tPSA and f/tPSA was proven to be effective in increasing the detection rate of prostate cancer. Many studies have demonstrated the excellent ability of PIRADS in predicting csPCa [12, 36, 37]. van Leeuwen et al. [33] carried out a multicenter retrospective study, whose results showed the promising effectiveness of a nomogram model constructed based on variables such as PIRADS score in identifying csPCa.

In addition, ethnic factor is also an important risk factor for the development of prostate cancer. In a genome-wide study of Chinese prostate cancer patients, 2 SNPs (9q31.2 and 19q13.4) were found to be strongly associated with prostate cancer prevalence in the Chinese population, which is significantly various from the genetic susceptibility to prostate cancer in European and American populations [38]. Another study based on 25,517 kidney cancer patients showed a significantly higher rate of postradical kidney surgery complications in blacks compared to whites [39]. Nevertheless, due to the limitations of the accumulated clinical data in this paper, the failure to include racial differences as an important factor in the study somewhat affects the accuracy of the predicted model.

There are several drawbacks in this study. First, this is a retrospective analysis. Thus, some selective biases may be unavoidable. Second, AISI is a nonspecific tumor marker, and further prospective randomized controlled trials are needed to validate our findings. Finally, blood cell parameters may be influenced by factors such as diet and genetic disorders, and we cannot completely exclude these factors.

## 5. Conclusion

In this study, we developed a nomogram model to predict the risk of csPCa and demonstrated its good predictive performance. This convenient tool would be helpful for clinicians in assessing the necessity of prostate biopsy in patients.

## Figures and Tables

**Table 1:** Baseline characteristics of the training and the validation cohort.

Variables	Training cohort (*n* = 853)	Validation cohort (*n* = 366)	*P* value
Age (y)	69 (63, 75)	68 (63, 74)	0.462
BMI (kg/m^2^)	23.66 (21.70, 25.66)	24.11 (21.63, 25.95)	0.239
AISI	230.77 (154.43, 367.47)	245.91 (145.79, 355.19)	0.851
SII	509.16 (362.76, 721.68)	508.46 (368.64, 752.96)	0.764
NLR	2.46 (1.90, 3.30)	2.42 (1.94, 3.52)	0.556
PLR	132.24 (105.20, 167.40)	131.03 (101.72, 174.51)	0.645
tPSA (ng/ml)	11.23 (6.40, 25.10)	10.99 (7.18, 23.85)	0.294
fPSA (ng/ml)	1.42 (0.85, 3.40)	1.62 (0.81, 3.19)	0.763
f/tPSA	0.13 (0.08, 0.19)	0.13 (0.08, 0.20)	0.653
PV (ml)	41.93 (28.97, 60.88)	43.78 (30.21, 64.05)	0.241
HBP			0.031
No	493 (57.8%)	187 (51.1%)	
Yes	360 (42.2%)	179 (48.9%)	
DM			0.920
No	773 (90.6%)	331 (90.4%)	
Yes	80 (9.4%)	35 (9.6%)	
PIRADS score			0.681
1	40 (4.7%)	18 (4.9%)	
2	157 (18.4%)	72 (19.7%)	
3	189 (22.2%)	84 (23.0%)	
4	260 (30.5%)	96 (26.2%)	
5	207 (24.3%)	96 (26.2%)	

**Table 2:** Descriptive characteristics of patients with no PCa and PCa.

Variables	No PCa (*n* = 398)	PCa (*n* = 455)	*P* value
Age (y)	66 (60, 72)	72 (66, 76)	<0.001
BMI (kg/m^2^)	23.52 (21.67, 25.41)	23.89 (21.72, 25.81)	0.087
AISI	188.83 (120.64, 285.10)	271.83 (186.04, 420.42)	<0.001
SII	482.46 (340.90, 695.39)	534.35 (382.67, 750.57)	0.009
NLR	2.36 (1.82, 3.20)	2.53 (1.96, 3.37)	0.023
PLR	130.97 (104.18, 166.11)	133.33 (107.73, 169.75)	0.385
tPSA (ng/ml)	8.45 (5.43, 12.50)	19.20 (9.08, 47.78)	<0.001
fPSA (ng/ml)	1.18 (0.74, 2.05)	2.05 (1.02, 5.90)	<0.001
f/tPSA	0.16 (0.10, 0.23)	0.11 (0.07, 0.16)	<0.001
PV (ml)	46.62 (33.80, 66.50)	35.94 (26.43, 54.40)	<0.001
HBP			0.268
No	238 (59.8%)	255 (56.0%)	
Yes	160 (40.2%)	200 (44.0%)	
DM			0.210
No	366 (92.0%)	407 (89.5%)	
Yes	32 (8.0%)	48 (10.5%)	
PIRADS score			<0.001
1	30 (7.5%)	10 (2.2%)	
2	130 (32.7%)	27 (5.9%)	
3	125 (31.4%)	64 (14.1%)	
4	97 (24.4%)	163 (35.8%)	
5	16 (4.0%)	191 (42.0%)	

**Table 3:** Descriptive characteristics of patients with no csPCa and csPCa.

Variables	No csPCa (*n* = 477)	csPCa (*n* = 406)	*P* value
Age (y)	66 (60, 72)	72 (66, 77)	<0.001
BMI (kg/m^2^)	23.53 (21.67, 25.49)	23.88 (21.80, 25.82)	0.111
AISI	195.65 (127.02, 292.69)	277.45 (187.15, 423.85)	<0.001
SII	485.47 (345.15, 703.70)	534.74 (384.76, 744.21)	0.017
NLR	2.38 (1.84, 3.24)	2.53 (1.98, 3.34)	0.044
PLR	131.88 (104.39, 166.03)	132.56 (107.47, 170.82)	0.603
tPSA (ng/ml)	8.64 (5.44, 12.57)	20.79 (10.21, 54.76)	<0.001
fPSA (ng/ml)	1.18 (0.75, 2.05)	2.25 (1.09, 6.30)	<0.001
f/tPSA	0.15 (0.10, 0.22)	0.11 (0.07, 0.15)	<0.001
PV (ml)	46.30 (33.02, 66.06)	35.83 (26.18, 53.86)	<0.001
HBP			0.612
No	262 (58.6%)	231 (56.9%)	
Yes	185 (41.4%)	175 (43.1%)	
DM			0.492
No	408 (91.3%)	365 (89.9%)	
Yes	39 (8.7%)	41 (10.1%)	
PIRADS score			<0.001
1	31 (6.9%)	9 (2.2%)	
2	141 (31.5%)	16 (3.9%)	
3	142 (31.8%)	47 (11.6%)	
4	112 (25.1%)	148 (36.5%)	
5	21 (4.7%)	186 (45.8%)	

**Table 4:** Logistic regression analysis for the risk factors in the training cohort.

Variables	Univariate analysis		*P* value	Multivariate analysis		*P* value
	OR	95% CI		OR	95% CI	
Age	1.090	1.070~1.111	<0.001	1.091	1.064~1.120	<0.001
tPSA	1.068	1.053~1.084	<0.001	1.037	1.009~1.067	0.010
fPSA	1.223	1.154~1.297	<0.001	1.027	0.878~1.201	0.742
f/tPSA	0.003	0.001~0.015	<0.001	0.009	0.000~0.176	0.002
AISI	1.002	1.001~1.003	<0.001	1.001	1.000~1.002	0.037
SII	1.000	1.000~1.001	0.315	—	—	—
NLR	1.068	0.964~1.183	0.206	—	—	—
PV	0.986	0.981~0.991	<0.001	0.978	0.970~0.986	<0.001
HBP	1.073	0.817~1.408	0.612	—	—	—
DM	1.175	0.741~1.863	0.492	—	—	—
PIRADS score			<0.001			<0.001
1	—	—	—	—	—	—
2	0.391	0.158~0.996	0.042	0.312	0.114~0.857	0.024
3	1.140	0.506~2.568	0.752	1.039	0.424~2.548	0.934
4	4.552	2.083~9.946	<0.001	2.766	1.157~6.616	0.022
5	30.508	12.800~72.712	<0.001	9.906	3.683~26.643	<0.001

**Table 5:** ROC curve comparing base model with base model with AISI and PIRADS score for csPCa.

Base model				Base model+AISI+PIRADS score			
AUC	SEN (%)	SPE (%)	*P* value	AUC	SEN (%)	SPE (%)	*P* value
0.861	80.3	80.5	<0.001	0.901	78.6	84.8	<0.001

Abbreviations: SEN: sensibility; SPE: specificity.

## Data Availability

The data used in this study are available from the corresponding author.
